# Efficacy of photochemical internalisation using disulfonated chlorin and porphyrin photosensitisers: An *in vitro* study in 2D and 3D prostate cancer models

**DOI:** 10.1016/j.canlet.2017.02.018

**Published:** 2017-05-01

**Authors:** Alejandra Martinez de Pinillos Bayona, Josephine H. Woodhams, Hayley Pye, Rifat A. Hamoudi, Caroline M. Moore, Alexander J. MacRobert

**Affiliations:** a Division of Surgery and Interventional Sciences, University College London, London, United Kingdom; b Department of Urology, University College London Hospital, London, United Kingdom; c College of Medicine, University of Sharjah, Sharjah, United Arab Emirates

**Keywords:** Photochemical internalisation (PCI), Photodynamic therapy (PDT), Prostate cancer, TPPS_2a_, TPCS_2a_

## Abstract

This study shows the therapeutic outcome of Photochemical Internalisation (PCI) in prostate cancer *in vitro* surpasses that of Photodynamic Therapy (PDT) and could improve prostate PDT in the clinic, whilst avoiding chemotherapeutics side effects. In addition, the study assesses the potential of PCI with two different photosensitisers (TPCS_2a_ and TPPS_2a_) in prostate cancer cells (human PC3 and rat MatLyLu) using standard 2D monolayer culture and 3D biomimetic model. Photosensitisers were used alone for photodynamic therapy (PDT) or with the cytotoxin saporin (PCI). TPPS_2a_ and TPCS_2a_ were shown to be located in discrete cytoplasmic vesicles before light treatment and redistribute into the cytosol upon light excitation. PC3 cells exhibit a higher uptake than MatLyLu cells for both photosensitisers. In the 2D model, PCI resulted in greater cell death than PDT alone in both cell lines. In 3D model, morphological changes were also observed. Saporin-based toxicity was negligible in PC3 cells, but pronounced in MatLyLu cells (IC50 = 18 nM). In conclusion, the study showed that tumour features such as tumour cell growth rate or interaction with drugs determine therapeutic conditions for optimal photochemical treatment in metastatic prostate cancer.

## Highlights

• The efficacy of PCI surpasses that of PDT *in vitro*. PCI could improve prostate cancer treatment and minimise side effects.
• 3D model observations confirm findings in previous 2D PCI investigations.
• Tumour features (i.e. doubling rate, interaction with drugs) will determine conditions for optimal photochemical treatment.

## Introduction

Prostate cancer is the most common type of cancer affecting males and 4th leading cause of death from cancer [1]. Radical therapies can have significant side effects, especially in terms of incontinence, sexual function, and bowel problems. In addition, a randomised study of men with localised prostate cancer showed no significant difference in overall or prostate cancer specific mortality between men who underwent radical surgery or observation only [2]. There is great interest therefore in the development of focal treatments which can treat the more significant cancers whilst sparing healthy prostate tissue.

Both Photodynamic Therapy (PDT) and Photochemical Internalisation (PCI) are light-based focal therapies that use photosensitisers (PS) which upon interaction with molecular oxygen, and irradiation with light of a specific wavelength, induce generation of reactive oxygen species (ROS). These will act on organic structures, lipid membranes, cellular organelles, ultimately causing cell death. In contrast, the low PS and light dose used in PCI treatment are designed to be sub-lethal since disruption of endolysosomal membranes is not particularly cytotoxic. The goal of PCI therefore is to enable the release of drugs that have been endocytosed and entrapped in such intracellular compartments so that they are not subject to degradation in mature lysosomes and can reach their intended intracellular target [3]. To achieve this, PCI requires amphiphilic photosensitisers which can localise in the endosomal membrane after being internalised by cells via adsorptive endocytosis so that a photooxidative effect can be exerted in the membrane, leading to its rupture [4], [5], [6]. Features of both TPPS_2a_ and TPCS_2a_ fulfil these requirements.

A number of different chemotherapeutic/photosensitiser combinations have been assessed in preclinical and clinical studies for a range of different cancers [3], [4], [7]. The ribosome inactivating proteins (RIP) type 1 toxins, gelonin and saporin, have been used as model therapeutics for PCI in several studies [8], [9], [10], [11], [12], [13], [14]. Both drugs have been efficiently delivered *in vitro* and *in vivo*, resulting in an improved outcome compared to PDT. Recently a first-in-man clinical trial has been published, successfully delivering bleomycin when combined with Amphinex^®^, which is based on TPCS_2a_, the same photosensitiser tested herein [15].

In the present study, the cytotoxic agent saporin was chosen for the PCI combination experiments. It is a large (30 kDa) RIP 1, which lacks the lectin B-chain that facilitates endocytosis of RIP 2 by cells such as ricin [16]. Therefore, despite the high enzymatic activity described for this family of enzymes, saporin is not able to interact efficiently with cytosolic ribosomes owing to endolysosomal sequestration.

PDT is currently being investigated for treatment of prostate cancer [17], [18], where focal illumination of tumour is achieved using an implanted array of fibre-optic catheters coupled to a laser of the appropriate wavelength. However, laser light-induced damage to nerves, urethra, rectum and urinary sphincter may still occur [19], [20]. The first-in-man clinical PCI dose-escalation study published in 2016 [15] indicated enhanced tumour selectivity of PCI over PDT for head and neck tumours, most likely due to lack of significant adverse effects in PCI. If these findings could be replicated in the prostate, side effects could be further ameliorated. Since the technology required for PCI is very similar to PDT, apart from the addition of a chemotherapeutic, PCI in the prostate should be technically feasible.

The aim of this study is to investigate the effect of PCI in prostate cancer *in vitro* using standard 2-dimensional (2D) and a 3-dimensional (3D) biomimetic collagen hydrogel that will mimic biological conditions more realistically [21]. In addition, disulfonated tetraphenyl porphyrin (TPPS_2a_) was compared to its chlorin analogue (TPCS_2a_). Both PS have two sulfonate groups substituted on adjacent phenyl rings which impart amphiphillic properties to these compounds, as required for PCI [3]. In our study, two prostate cancer cell lines were used: firstly, human PC3 cells which have high metastatic potential and have been used in advanced prostatic cancer studies [22]. Secondly, a rat line MatLyLu, which has previously been used for syngeneic tumour rat model studies [23], [24].

## Material & methods

### Cell lines and cell culture

PC3 (grade IV human prostate adenocarcinoma, androgen-independent) and MatLyLu (rat prostate carcinoma, androgen-independent). Both cell lines were routinely grown in RPMI 1640 containing l-glutamine, 10% Fetal Bovine Serum, 1% Penicillin-Streptomycin; at 37 °C, 5% CO2.

### Chemicals and drugs formulation

TPPS_2a_, tetraphenyl disulfonated porphyrin, Frontier Scientific Inc. US: a stock solution was prepared by dissolving the powder in DMSO. TPCS_2a_ was kindly donated by PCI Biotech AS (Oslo, Norway). Saporin (Sigma Aldrich) was dissolved in PBS. The molecular weights of the chlorin (MWT = 777) and porphyrin PS are essentially the same, with the chlorin (being a reduced porphyrin) having two more hydrogen atoms present on the macrocycle than the porphyrin. All drug solutions were administered in complete cell media, at 0.4 μg/ml and 2 nM.

### Conjugation of Alexa-Fluor488^®^ to Saporin and purification

Alexa-Fluor488^®^ was conjugated to Saporin according to a protocol from Molecular probes labelling kits (ThermoFisher Scientific, Cat. Number A 20000). Conjugate concentration was obtained using UV-visible absorbance measurements at 280 nm (Saporin) and 495 nm (*Alexa-Fluor488*^*®*^) in an ELX800 plate reader (BioTek Instruments, Inc., Bedfordshire, UK).

### Light source

PDT and PCI studies were conducted using a LumiSource^®^ (PCI Biotech, Norway), flat-bed lamp system composed of four fluorescence tubes with peak emission at 420 nm and 7 mW/cm^2^ output. Fluorescence redistribution studies followed on-stage illumination with an inverted fluorescence microscope equipped with a blue diode laser module at 405 nm.

### 2-Dimensional studies

Cells were seeded directly onto 96-well plates. PC3 cells were seeded at 10,000 or 5000 cells/well for 24 or 96 h following light treatment experiments; MatLyLu cells were seeded at 1000 or 600 cells/well for 24 or 48 h following light treatment experiments.

### Fabrication of the collagen 3-dimensional hydrogels

Gels were prepared using 80% v/v Type I rat tail collagen (2 mg/ml in 0.6% acetic acid) and mixed with 10% v/v Minimum Essential Medium (MEM) 10× (Sigma Aldrich). This solution was then neutralised using 1:10 and 1:100 dilutions of Sodium Hydroxide. The neutralised mixture was added to 10% v/v cell suspension. 100 μl of the mixture was added to individual wells of 96-well plates. The well plates were incubated for 5 min at 37 °C and 5% CO_2_ for collagen to gel; culture media was then added.

### Light treatment of TPPS_2a_, TPCS_2a_ and saporin *in vitro*

Cells were incubated with a combination of either TPPS_2a_ or TPCS_2a_, with saporin for 24 h and then washed with PBS and fresh cell medium without the photosensitiser was added. Four hours later, excitation of photosensitisers was carried out for 3 or 5 min (1.3 and 2.1 J/cm^2^ respectively). All experimental procedures were carried out under low light conditions.

### Cytotoxic effects of photochemical internalization

*MTT assay [3-(4, 5-dimethylthiazolyl-2)-2, 5-diphenyltetrazolium bromide]* (Sigma Aldrich M2128) was used to assess viability. Cell media was replaced with a solution of 1 mg/ml MTT either at 24, 48 or 96 h after light treatment. The plates were then returned to the incubator for 1.5 h before dissolving formazan crystals in 100 μl DMSO. Absorbance at 570 nm was recorded using ELX800 plate reader (BioTek Instruments, Inc., Bedfordshire, UK).

### Viability staining

A LIVE/DEAD^®^ Cell Imaging Kit (488/570, Thermofisher Scientific) was used to assess cell death in 3D hydrogels. Viable cells relate to the conversion cell-permeant calcein AM to intensely green fluorescent calcein. Culture media was removed from the wells and gels were incubated with dead/live imaging kit for 15 min, washed three times in PBS and imaged and analysed using an Olympus Fluoview 1000 confocal laser-scanning microscope with Image J. Cell viability was observed comparing green fluorescence channel and transmitted light.

### Intracellular localisation of photosensitiser & Saporin-Alexa-Fluor488^®^

Both PC3 and MatLyLu cells were seeded onto glass bottom dishes FluoroDish™ (World Precision Instruments, Inc.) at 9000 cells/dish and 2000 cells/dish respectively.

Cells were incubated with TPPS_2a_ or TPCS_2a_ alone or combined with Saporin-Alexa-Fluor488^®^ for 24 h and then washed with PBS and fresh cell medium without the photosensitiser was added. A 75 nM solution of LysoTracker^®^ Red DND-99 in phenol red free cell media was added 30 min prior to microscope imaging. Four hours after washing off the drugs, fluorescence of Saporin-Alexa-Fluor488^®^ was imaged using an inverted Olympus Fluoview FV1000 confocal microscope using a 488 nm laser. Additionally, a 569 nm laser was used to image LysoTracker^®^ Red DND-99. Image analysis was performed with Fluoview FV1000 (Olympus) and Image J software.

### TPPS_2a_ & TPCS_2a_ uptake in PC3 & MatLyLu cells

PC3 and MatLyLu cells were seeded onto 96-well plates at a cell seeding density of 10000 cells/well or 1000 cells/well respectively and incubated for 24 h with increasing doses of either TPPS_2a_ or TPCS_2a_ (0.2–0.8 μg/ml). Plates were then washed once with PBS and phenol red free fresh cell media was added into the wells. Fluorescence emission was measured using a LS50B Perkin–Elmer spectrofluorimeter (Perkin–Elmer, Beaconsfield, UK), exciting at 420 nm and detecting at 650 nm.

### Fluorescence microscopy of TPPS_2a_ and TPCS_2a_

Subcellular localisation and redistribution of photosensitiser molecules upon light administration was assayed using an Olympus IMT-2 epi-fluorescence inverted microscope (20× magnification objective, 250 × 250 micron scale). PC3 and MatLyLu cells were seeded onto glass bottom dishes FluoroDish™ (World Precision Instruments, Inc.) at 9000 cell/dish and 2000 cell/dish respectively. A 24-h attachment and growth period was allowed before incubating the cells for 24 h with either TPPS_2a_ or TPCS_2a_ in culture media. Dishes were then washed off once with PBS and phenol red free media was added.

Cell recovery was allowed for a further 4-h period prior to irradiation of photosensitisers (0.35 μg/ml TPPS_2a_ or TPCS_2a_) “on stage” using a 2 mW 405 nm blue diode laser module (Thorlabs Inc.) coupled to a liquid light guide. The microscope was attached to a 512 × 512 pixel cooled charge-coupled device (CCD) camera (PIXIS 512F, Princeton Instruments Inc.), used to record fluorescence images using a 660 nm bandpass detection filter (Omega Optical Inc.). Short exposure times of 2 s were used to record nascent fluorescence images. Images were obtained at different time-points following further on-stage irradiation to image the photo-induced redistribution of TPPS_2a_ or TPCS_2a_.

### Statistical analysis

Experiments carried out in 96-well plates were averaged across 16 wells and performed in triplicate. Data were analysed using two-way ANOVA and Bonferroni *post hoc* multiple comparison testing using Prism software version 6. Error bars from the mean show +/− standard deviation (SD). A minimum significance level of P < 0.05 was used for all statistical tests.

To test for a synergistic interaction between the two separate therapies applied, we used the following equation:

$$\alpha =\frac{\left[\%\text{V}\left(\text{PDT}\right)\times \;\%\text{V}\left(\text{cytotoxin}\right)\right]}{\%\text{V}\left(\text{combination}\right)}$$

where in the numerator %V is the percentage viability for each separate therapy (i.e. PDT and the application of the cytotoxin), and % V in the denominator is the percentage viability observed following the PCI combination treatment [25], [26]. If α > 1 then a synergistic effect has been observed whereas an antagonistic effect is denoted by α < 1. Τhis analysis has been used previously by others to identify synergistic effects in PCI [26].

## Results

### Photosensitiser uptake in PC3 and MatLyLu cells

**Fig. 1:**
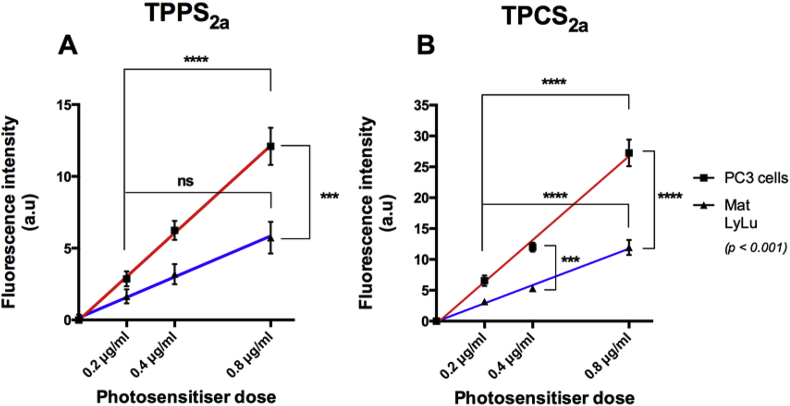
**Uptake of TPPS**_**2a**_**& TPCS**_**2a**_**in PC3 and MatLyLu cells**. PC3 and MatLyLu cells were exposed to increasing doses of photosensitiser (0.2 μg/ml – 0.8 μg/ml) during 24 h and uptake was related to fluorescence emission.

The intensity of characteristic photosensitiser fluorescence was used as an estimate of intracellular uptake. Cellular fluorescence in PC3 and MatLyLu whole-cells increased with the PS dose in both cell lines, and a linear dose-dependency for both photosensitisers in PC3 and MatLyLu cells was seen (Fig. 1). After 24 hr-exposure to increasing concentrations of drug solutions, uptake was up to 2.3-fold higher in PC3 than MatLyLu cells (Fig. 1). Significant differences were also found between the lowest and highest doses in nearly all cases within each cell line (p < 0.001). Comparing the fluorescence levels for the photosensitisers in each cell line, administration of TPCS_2a_ yielded higher fluorescence in both cell lines by approximately a factor of two compared to TPPS_2a_ at the same dose [25].

### Comparison of the effect of PDT and PCI on prostate cancer cell viability in a 2D environment

**Fig. 2:**
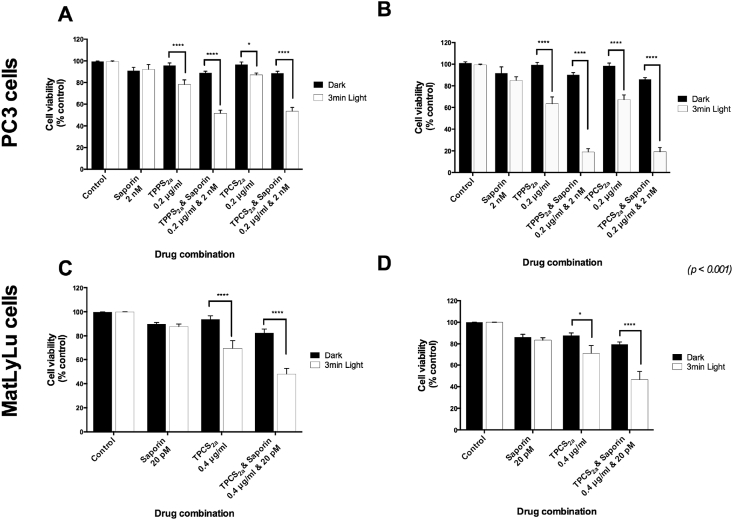
**Reduction of Cell viability of PC3 and MatLyLu cells upon exposure to PDT & PCI**. (A, B) Relative cell viability of PC3 cells following incubation to 0.2 μg/ml TPPS_2a_ or TPCS_2a_ alone (PDT) or combined with Saporin 2 nM (PCI) during 24 h and posterior illumination during 3 min. Cell viability was measured through the MTT assay either 24 h (A) or 96 h (B) after photosensitiser light-excitation. (C, D) Relative cell viability of MatLyLu cells following incubation to 0.4 μg/ml TPCS_2a_ alone (PDT) or combined with Saporin 20 pM (PCI) during 24 h and posterior illumination during 3 min (1.3 J/cm^2^). Cell viability was measured through the MTT assay either 24 h (C) or 48 h (D) after photosensitiser light-excitation.

A significant reduction of cell viability (p < 0.001) was observed when human PC3 and rat MatLyLu prostate cancer cells were treated with PDT, regardless of the photosensitiser used (Fig. 2). In the rat model cell viability was measured up to 48 h after light treatment (Fig. 2D). Furthermore, the difference between cell death after PDT and PCI was synergistic between PDT and saporin (Fig. 2A–D).

Comparing PCI and PDT at 96 h (see Table 1) there is a 3-fold enhancement in cytotoxicity - for PDT the viability is 64% and for PCI the viability is down to 19% for TPCS_2a_-even though saporin alone only elicits a 10% reduction in cytotoxicity. Using the equation for assessing synergy described in Materials & Methods, values of α were calculated as 2.9 and 3 at 96 h for TPPS_2a_ and TPCS_2a_. These values exceed unity which is consistent with a synergistic combined therapeutic response which we attribute to PCI. Using 24 h to measure viability the difference is less pronounced yet still significant, with α at 1.4 and 1.5 for TPPS_2a_ and TPCS_2a_.

The MatLyLu cells showed much higher sensitivity to saporin alone than PC3 cells, so we therefore reduced the saporin dose in order to keep the dose sub-lethal (Fig. 2C and D). Due to the quicker MatLyLu cell doubling time compared to PC3 cells, seeding was restricted to 48 h post-light to ensure that the MTT viability assay was useable. Photosensitiser doses for the MatLyLu cells were higher than that for the PC3 cells to achieve a comparable effect. As with the PC3 cells, PCI resulted in greater cell death compared to PDT, by a factor of 3.5 when measured 96 h after light treatment (Fig. 2B), giving an α value of 1.3 at 24hr.

In addition, cell killing seen between 24 and 96 h in PC3 cells was significantly higher in PCI than PDT groups (Fig. 2A and B). Both photosensitisers showed a similar biological activity in PC3 cells (see summary Table 1). The effect of PDT and PCI in a 3D model was then investigated.

### Comparison of the effect of PDT and PCI on prostate cancer cells seeded in a biomimetic 3D models

**Fig. 3:**
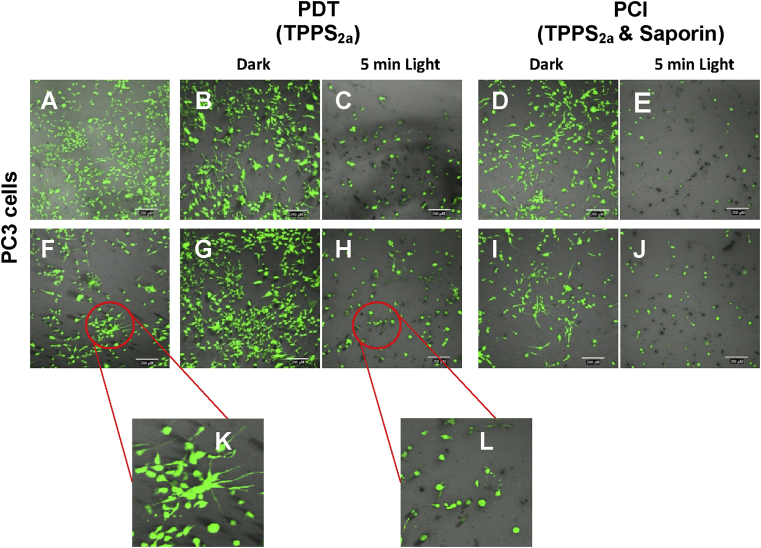
**PDT & PCI in PC3 cells seeded in 3D collagen hydrogels**. Fluorescence microscopy of PDT and PCI treated PC3 cells in 3D cultures. (A) PC3 control cells. (B) TPPS_2a_ (0.2 μg/ml), 96 h after no light conditions. (C) TPPS_2a_ (0.2 μg/ml) 96 h after 5 min light (2.1 J/cm^2^) (PDT). (D) TPPS_2a_ (0.2 μg/ml), Saporin (2 nM), 96 h after no light conditions. (E) TPPS_2a_ (0.2 μg/ml), Saporin (2 nM), 96 h after 5 min light (2.1 J/cm^2^) (PCI). (F) Saporin (2 nM), (K) higher magnification of (F). (G) TPCS_2a_ (0.2 μg/ml), 96 h after no light conditions. (H) TPCS_2a_ (0.2 μg/ml) 96 h after 5 min light (2.1 J/cm^2^) (PDT), (L) higher magnification of (H). (I) TPCS_2a_ (0.2 μg/ml), Saporin (2 nM), 96 h after no light conditions. (J) TPCS_2a_ (0.2 μg/ml), Saporin (2 nM), 96 h after 5 min light (2.1 J/cm^2^) (PCI). Scale bars shown as 200 μm.

Cell viability in collagen hydrogels was observed staining with calcein to assess for cell viability through the emission of green fluorescence; unstained dead cells appear as black spots (Fig. 3). In keeping with the results from our 2D *in vitro* experiments, we found a significant reduction of cell viability in cells treated with PDT (Fig. 3C and H) and PCI (Fig. 3E and J). PCI resulted greater cell death than PDT, regardless of which photosensitiser was used (Fig. 3E and J). In addition, changes in cell morphology were seen after exposure to light (Fig. 3L). Non PDT- or PCI- treated cells appear as elongated (Fig. 3A, B, D, F, G and I) as opposed to treated cells (Fig. 3C, E, H and J) which adopted a rounded shape, suggesting non-viability (see detailed Fig. 3K and L).

Based on the findings from both 2D and 3D cell viability studies, the results show that PCI effect is superior to that exerted by PDT, as the combination of saporin and photosensitiser is synergistic (Fig. 2).

### Subcellular localisation of TPPS_2a_ and TPCS_2a_ before and after light treatment

**Fig. 4:**
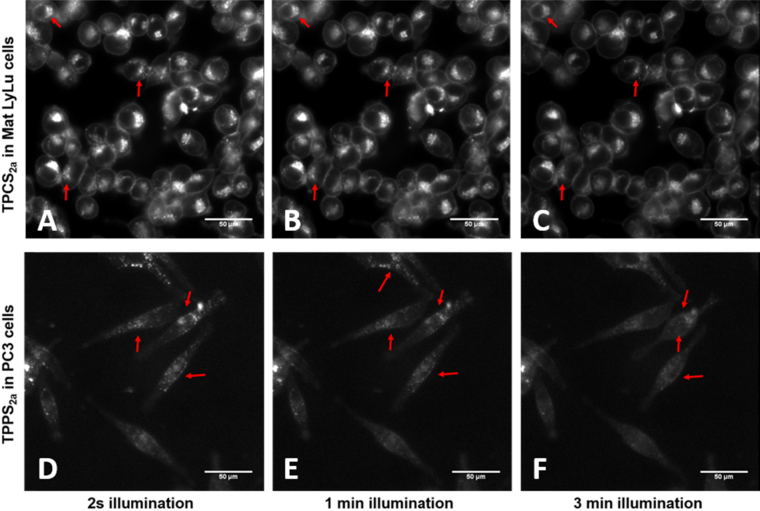
**Subcellular localisation of TPPS**_**2a**_**& TPCS**_**2a**_**in MatLyLu and PC3 cells**. Optical microscopy of TPPS_2a_ & TPCS_2a_ in PC3 and MatLyLu cells. (A) TPCS_2a_ (0.35 μg/ml) in MatLyLu cells prior to light-excitation. (B) TPCS_2a_ (0.35 μg/ml) in MatLyLu cells 3 min post light-excitation. (C) TPPS_2a_ (0.35 μg/ml) in PC3 cells prior to light-excitation. (D) TPPS_2a_ (0.35 μg/ml) in PC3 3 min post light-excitation. Scale bars shown as 50 μm.

TPPS_2a_ and TPCS_2a_ appear as discrete granules in the cytoplasm when endocytosed by either PC3 or MatLyLu cells (highlighted with red arrows in Fig. 4), consistent with the endolysosomal localisation required for PCI. Exposure of cells to light for 2 s needed to acquire these images was not sufficient to perturb the nascent distribution of photosensitisers and lead to reduction–oxidation reactions characteristic of PDT and consequently PCI (Fig. 4A and D).

However, upon longer illumination (shown up to 3 min), relocalisation of these cytosolic structures was seen (Fig. 4C and F). We interpret this as a photo-induced disruption of endolysosomes where photosensitisers are initially localised, into the cytosol which results in a more diffuse intracellular fluorescence pattern. The intracellular dispersal of the fluorescence also leads to a lower apparent intensity or ‘dissipation’ of the fluorescence. Intermediate micrographs taken 1 min post illumination (Fig. 4B and E) show the beginning of the redistribution process, where both intact and disrupted vesicles co-exist, inconsistent with a photobleaching process.

Despite the comparable uptake of TPPS_2a_ in PC3 and TPCS_2a_ in MatLyLu cells (Fig. 1), the latter resulted in significantly brighter fluorescence (Fig. 4A and B). This greater intensity could also be due to the higher fluorescence efficiency yield [27].

### Saporin-Alexa-Fluor488^®^ subcellular localisation & redistribution upon light excitation

**Fig. 5:**
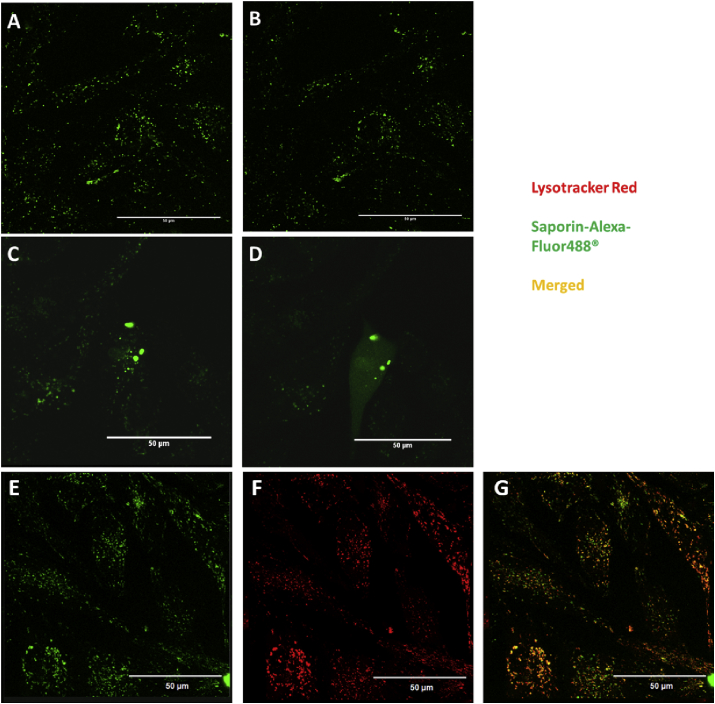
**Saporin-Alexa-Fluor488**^**®**^**subcellular localisation & redistribution upon excitation of TPPS**_**2a**_. Fluorescence microscopy of Saporin-Alexa Fluor488^®^ in PC3 cells. (A) Saporin-Alexa Fluor488^®^ (400 nM) prior to illumination with 405 nm laser. (B) Saporin-Alexa Fluor488^®^ (400 nM) post exposure to 405 nm laser. (C) Saporin-Alexa Fluor488^®^ (400 nM) and TPPS_2a_ (0.4 μg/ml) prior to illumination with 405 nm laser. (D) Saporin-Alexa Fluor488^®^ (400 nM) and TPPS_2a_ (0.4 μg/ml) post exposure to 405 nm laser. (E) Saporin-Alexa Fluor488^®^ (400 Nm). (F) LysoTracker^®^ Red DND-99 (75 nM). (G) Merged Saporin-Alexa Fluor488^®^ (E) & LysoTracker^®^ Red DND-99 (F). Scale bars shown as 50 μm.

In the present study, we administered saporin labelled with the fluorescent dye Alexa-Fluor488^®^ either alone or combined with TPPS_2a_. Upon uptake in PC3 cells, Saporin-Alexa-Fluor488^®^ forms discrete cytosolic vesicles similar to the above described for TPPS_2a_ in PC3 and TPCS_2a_ in MatLyLu (Fig. 5A, C and E). Illumination with a 405 nm laser for 1 min showed dispersal of the fluorescence only if the labelled toxin was combined with the mentioned photosensitiser (Fig. 5D). The absorption of both porphyrins and chlorins is characterised by a very intense band around 400 nm (Soret band) [27]; therefore, we would expect efficient excitation of TPPS_2a_ upon exposure to the 405 nm laser. Some co-localisation was found between the green fluorescence of Alexa-labelled saporin located in cytosolic compartments (Fig. 5E) and lysosomes (Fig. 5F), using co-excitation of lysotracker red (Fig. 5G). Subcellular localisation and redistribution of labelled saporin when co-administered with TPCS_2a_ were also observed (data not shown).

## Discussion

Previous *in vitro* studies have compared PCI-induced cytotoxicity using a variety of drugs and photosensitisers with the purely photooxidative effect caused by PDT [9], [28], [29], [30]. PCI has also recently been tested in a range of human and non-human bladder cancer cell lines [31] with a view for using PCI for treatment of tumours in the bladder, which underlines the interest in urological applications of PCI. A study on the uptake of TPCS_2a_ in orthotopic bladder tumours in rats has also been carried out, prior to PCI studies [32]. As regards prostate cancer, very few studies have been conducted on PCI involving either androgen dependent cells (LnCaP) [12] or androgen independent cells with moderate metastatic potential (DU145) [12], [33], [34]. Our study is the first report on PCI-treated highly metastatic, androgen independent prostate cancer models, PC3 and MatLyLu. The latter will pave the path for ongoing investigations in a rodent model.

The examination of the efficacy of PCI in our prostate carcinoma cells for delivery of saporin showed a considerable enhancement in cytotoxicity, especially human PC3 cells in the 2D model. We used the RIP 1 inhibitor saporin as a model chemotherapeutic agent. Although it is relatively large, it does serve as a basis for comparison with bleomycin which was used in the clinical study, and larger chemotherapeutics including nanomedicines which are all prone to endolysomal entrapment and degradation, for which PCI is potentially suitable. Our results demonstrated that PCI in PC3 cells elicits a significantly therapeutic outcome compared to that exhibited by PDT (increase by 3.5-fold). With the MatLyLu cells, PCI was less effective, possibly owing to more efficient uptake of saporin as discussed below, although synergicity between PDT and saporin was still present (Fig. 2).

The photosensitisers exhibited comparable efficacy for PCI, which is not surprising given their near identical molecular structure. *In vivo* treatment where red light would be used, would strongly favour the chlorin which exhibits a much stronger red absorption than the corresponding porphyrin.

Interestingly, the intrinsic toxicity of saporin was considerably more toxic in MatLyLu cells than PC3. The much shorter doubling time of the MaLyLu cells suggests higher membrane turnover, and thus increased endocytosis which could explain the higher relative efficacy of saporin. Another possibility is that saporin's uptake in MatLyLu cells could occur independently of the B chain known to facilitate entry of RIP type II, i.e. ricin [35], [36].

Optimal performance of PCI requires keeping both the PDT effect at sub-threshold lethality and likewise for the chemotherapeutic. Consequently, the saporin dose was reduced in the rat cell line, whereas TPCS_2a_ dose was increased since a 2-fold lower amount of either TPPS_2a_ or TPCS_2a_ was found in MatLyLu cells than PC3 cells 24 h after administration (Fig. 1). The linear dependence of cellular fluorescence vs. dose indicates that the presence of intracellular aggregation of both photosensitisers, which will be weakly or non-fluorescencent and will form at higher applied doses, is minimal in each cell line for this dose range [10]. The relative gradient of the fluorescence intensity versus dose is approximately two-fold higher for TPCS_2a_ vs. TPPS_2a_ for each cell line. However although a higher TPCS_2a_ fluorescence intensity was observed in both lines, the relative fluorescence quantum yield of each PS needs to be taken into consideration when assessing relative cellular concentrations. Lilletvedt et al. [27] concluded that TPCS_2a_ is a more efficient fluorophore than the porphyrin counterpart in all the solvents studied: for example in ethylene glycol, the fluorescence quantum yield of TPCS_2a_ is 0.3 compared to 0.13 for TPPS_2a_, i.e. nearly a factor of two higher for the chlorin. Therefore assuming that the same trend applies in the cellular environment with the chlorin yielding higher fluorescence than the porphyrin for the same concentration, our results suggest that the relative cellular concentrations of both compounds in MatLyLu and PC3 cells are comparable (Fig. 1).

Fluorescence imaging confirmed intracellular redistribution of TPPS_2a_ and TPCS_2a_ following the application of light which is consistent with endolysosomal rupture in both cell lines (Fig. 4). Light-induced redistribution and cytosolic delivery of saporin using fluorescently labelled saporin, initially present in lysosomes, was also confirmed (Fig. 5). The fluorescence dispersal post-illumination data are consistent with photo-induced oxidative damage to endolysosomal membranes required for PCI [6].

Our experiments *in vitro* showed a good correlation between the standard 2D model and the 3D collagen hydrogel model. In addition to observing the effect on cell viability after PDT or PCI treatment, the 3D model experiments shed light on how cellular morphology is affected by the treatment (Fig. 3) probably mimicking what occurs *in vivo*. Aside from the decrease in the amount of viable cells after light treatment, the remaining cells lost their elongated phenotype and acquired a rounded morphology, also consistent with imminent cell death. Such information can be used for optimising PCI to treat surgical margins. Previous drug testing has shown discrepancies between 2D and *in vivo* studies, which has led to interest in the development of biomimetic 3D cancer models as a means to screen drugs [21] since cells can grow and interact in a more physiologically relevant environment.

In summary, firstly, based on findings in both 2D and 3D cell viability studies, we confirm that the PCI effect is superior to the one exerted by PDT, owing to the synergistic combination of saporin and photosensitiser with light. Moreover, we have demonstrated the efficacy of PCI for treatment of prostate cancer cells. A recent clinical trial on PDT using Padeliporfin as the photosensitiser reported encouraging results for treating low risk prostate cancer which demonstrates the feasibility of focal treatment of the prostate cancer using photosensitisers [37]. Secondly, our 3D experiments confirm the reliability of previous observations in published 2D studies. Finally, this study focuses on highly metastatic and aggressive cancer models indicating the potential of both PDT and PCI in more challenging tumours. Further studies will be required *in vivo* to demonstrate translational potential.

## Figures and Tables

**Table 1:** Summary of cell viability after PDT & PCI in 2D models *in vitro*.

PC3 cells				MatLyLu cells			
**24 h**	Saporin	90% ± 9		**24 h**	Saporin	88% ± 4.7	
	TPPS_2a_	PDT	78% ± 12.4		TPCS_2a_	PDT	70% ± 16.8
		PCI	52% ± 7.5				
	TPCS_2a_	PDT	87% ± 5			PCI	48% ± 10.6
		PCI	54% ± 7.8				
**96 h**	Saporin	85% ± 8.3		**48 h**	Saporin	83% ± 5	
	TPPS_2a_	PDT	64% ± 13.8		TPCS_2a_	PDT	71% ± 19.2
		PCI	19% ± 6.6				
	TPCS_2a_	PDT	67% ± 9.9			PCI	47% ± 16.5
		PCI	19% ± 8.7				
